# Evaluating the Integration of Patient Safety in Medical Training in Spain

**DOI:** 10.3389/ijph.2024.1607093

**Published:** 2024-04-25

**Authors:** Jesús María Aranaz Andrés, Marco Antonio Espinel Ruiz, Luis Manzano, Fernando De Jesus Franco

**Affiliations:** ^1^ Department Preventive Medicine and Public Health, Ramón y Cajal University Hospital, Madrid, Spain; ^2^ Faculty of Health Sciences, Universidad Internacional de La Rioja, Logroño, La Rioja, Spain; ^3^ Ramón y Cajal Institute for Health Research, Madrid, Spain; ^4^ Doctoral Program Health Science in Doctoral School of the University of Alcalá, Alcalá de Henares, Madrid, Spain; ^5^ Department Internal Medicine, Ramón y Cajal University Hospital, Madrid, Spain; ^6^ Department of Medicine and Medical Specialities, School of Medicine and Health Sciences, University of Alcalá, Alcalá de Henares, Spain

**Keywords:** patient safety, education, medical faculties, curse syllabus, patient safety topics

## Abstract

**Objectives:** The aim of this study was to determine the degree of integration of patient safety in the training of medical faculties at universities in Spain.

**Methods:** A descriptive, cross-sectional study was conducted. An assessment was made of the curse syllabi of Spanish medical schools, summarizing the proportion of faculties that present each of the topics recommended in the WHO’s curriculum guide.

**Results:** Of the 49 faculties, access to the curse syllabus of the subjects for the academic year 2023-2024 was obtained from 38 (78%). Although 82% of the faculties integrated some patient safety topic, only 56% included between 1 and 3 of the 11 topics recommended by WHO. The maximum number of integrated topics was 7, and this was only achieved by 1 faculty.

**Conclusion:** There is progress in the incorporation of fundamental concepts in patient safety, but the comprehensive implementation of all topics recommended by the WHO in Spanish medical schools is insufficient.

## Introduction

The World Health Organization (WHO) defines Patient Safety (PS) as the discipline of healthcare aiming to prevent, reduce risks, errors, and harm to patients during the provision of healthcare services [1, 2]. Safety incidents affect not only patients and their families but also the involved healthcare staff [3].

Since the United States Institute of Medicine issued the report “To Err is Human” in 1999, awareness and importance of patient safety have been increasing [4]. Since then, plans and strategies have been developed to improve patient safety at the international level. One of these strategies is to incorporate patient safety into the training of healthcare personnel.

In 2021, at the 74th World Health Assembly, the WHO approved the Global Patient Safety Action Plan 2021–2030 [1], one of its primary objectives is the importance of patient safety education, promoting the participation of multisectoral institutions, including universities. For this purpose, continuous improvement is necessary, supported by various PS tools [5]. Higher or university education is an essential environment in the transformation of society [6, 7]. Health education must ensure that graduates have achieved competencies, skills, values, and attitudes that enable them to meet the challenges their positions demand [8, 9].

In various countries such as the United States [7, 10], the United Kingdom [11, 12], Australia, and Canada [13], recommendations have been developed to improve training in patient safety. In Japan, in 2008, the Ministry of Education, Culture, Sports, Science, and Technology revised the medical curriculum to include patient safety as an important part of education [14, 15]. In Spain, although the Ministry of Health promoted the Patient Safety Strategy of the National Health System since 2005, identifying the need to create training and a culture of patient safety among health personnel [5], and in 2008 it was published in the BOE (State Official Newsletter) that among the competencies that medical students must acquire are “the evaluation of care quality and patient safety strategies” [16], little progress has been made in the basic training of health personnel [17]. Most of the patient safety training actions are focused on continuing education in postgraduate studies (Training in Patient Safety and Prevention of Adverse Events in Healthcare [18]; Training in Risk Management and Improvement of Patient Safety [19]), while in undergraduate studies, training depends more on the specific interest of a professor [20].

The WHO has published recommendations on how patient safety training should be implemented at universities [21]. This organization proposes 11 specific patient safety topics [21] and suggests various ways to provide training, from seminars to practice groups. Similarly, the National Health Service (NHS) of England has published documents about how this training should be implemented [11, 12].

In Europe, a published study [22] observed that 60% of the examined European faculties do not have any of the WHO-recommended patient safety topics integrated into their training. Other published studies that aim to measure the extent of undergraduate training in patient safety assess the level of knowledge and/or attitudes of students towards patient safety [17], evaluate the curriculum of a faculty [4], or through surveys, either of individual universities or at the national study [10, 23].

Neither in Spain nor in Latin America have studies been conducted to examine the integration of the topics recommended in the WHO’s patient safety curriculum guide into the training plans of medical faculties.

The curriculum is a document that contains basic information about each degree, while as defined by the National Agency for Quality Assessment and Accreditation (ANECA), the curse syllabus of a subject is the reference document for students and teachers, describing the objectives, contents, competencies to be acquired, evaluation methodology, bibliography of a subject [24].

The aim of this study was to determine the degree of integration of patient safety in the training of medical faculties at universities in Spain. The study examined the number of medical faculties that include the WHO-recommended patient safety topics in their curse syllabi. Finally, the integration of patient safety was evaluated based on its funding.

## Methods

### Study Design

This is a descriptive, cross-sectional study that involved an evaluation of the curse syllabus of medical all medical curses at various Spanish universities available on the official websites of each center. The objective was to assess whether the topics included in the WHO’s proposed curriculum guide were integrated into the medical students’ education.

### Study Population

The study population included medical faculties in Spain in 2023, which had an official website. The list of these universities was collected from the website of the Ministry of Universities. Faculties of medicine whose curse syllabi for the year were not available on their websites were excluded from the study.

### Data Collection

The data collection period was from April 2023 to August 2023.

Information available on the official websites of the medical faculties was accessed. The following information was collected: Public access to the curse syllabi of the faculty’s subjects, the Spain autonomous community to which the faculties belong, and the type of funding of the university (public or private).

A review of the subjects developed in the degree was conducted, focusing on those with clinical, surgical, preventive medicine, health management, or related.

A form was filled out for each medical faculty, recording the presence of the specific patient safety topics recommended in the WHO’s curriculum guide.

### Definitions

Integration of patient safety topics in the curse syllabus of any of the degree subjects: It was considered that one of the topics recommended in the WHO’s curriculum guide was integrated into the curse syllabus of a subject when explicit mention was made of patient safety. Therefore, when a subject addressed a topic without explicitly mentioning patient safety in the rest of the subject, it was considered not to be effectively integrated into the framework of training in patient safety. For example, the control of Health Care-Associated Infections (HCAIs) was not considered explicitly as part of the field of patient safety unless patient safety was specifically mentioned as a didactic topic in the subject where patient safety was mentioned.

The variables collected were the presence in any subject of the topics referred to in Table 1.

**TABLE 1:** Topics recommended in the World Health Organization patient safety curriculum guide [16]. (Geneva, Switzerland, 2009).

Topics recommended by the world health organization
What is patient safety?
What is human factors and why is it important to patient safety?
Understanding systems and the impact of complexity on patient care
Being an effective team player
Understanding and learning from errors
Understanding and managing clinical risk
Introduction to quality improvement methods
Engaging with patient and carers
Minimizing infection through improved infection control
Patient safety and invasive procedures
Improving medication safety

### Statistical Analysis

A descriptive analysis of the integration of patient safety in medical faculties was conducted, summarizing the proportion of faculties that present each of the topics, the proportion of faculties, and the number of patient safety topics they develop.

The association between university funding and the presence of training in patient safety was explored using Fisher’s exact test.

## Results

In the entire Spanish territory, a total of 49 medical faculties were identified, distributed across 16 autonomous communities (Supplementary Material S1). Of these, 13 belong to private university institutions, while 36 are under the management of public universities. The same table summarizes the web page address of each faculty, as well as the city and Spanish autonomous community to which they belong.

The Autonomous Community of Madrid stood out as the region with the highest concentration of medical faculties, hosting a total of 9 educational institutions of this type. Following were Catalonia, Andalusia, and the Valencian Community, with 8, 7, and 6 faculties, respectively.

When examining the type of funding for private medical faculties, it was observed that the autonomous Community of Madrid had the highest number of private faculties, with a total of 5. Catalonia and the Valencian Community followed, both with 2 private faculties each (Table 2).

**TABLE 2:** Public/private medical schools by autonomous community. (Spain, 2024).

Autonomous community	Number faculties	Public	Private
**Andalucia**	7	7	0
**Aragón**	1	1	0
**Ast** **urias**	1	1	0
**C. Madrid**	9	4	5
**C. Valenciana**	6	4	2
**Cantabria**	1	1	0
**Castilla La Mancha**	2	2	0
**Castilla y León**	2	2	0
**Cataluña**	8	6	2
**Extremadura**	1	1	0
**Galicia**	1	1	0
**Islas Baleares**	1	1	0
**islas Canarias**	3	2	1
**Murcia**	2	1	1
**Navarrra**	2	1	1
**Pais Vasco**	2	1	1
**Total**	**49**	**36**	**13**

In bold: Total number of faculties of medical school on all universities, public universities or private universities on all autonomous communities.

All faculties, both public and private, had a website hosting information about the medical degree program. Of the 49 faculties, access to the faculty’s curse syllabi for the year 2023-2024 was obtained from 38 (78%).

Regarding the 38 faculties where access to the curse syllabi was obtained, it was observed that no faculty implemented all the recommendations of the WHO’s patient safety curriculum guide, despite 14 years since its publication (Table 3). In addition, only 1 (3%) faculty developed 7 of the topics recommended by the curse syllabi, while 4 (11%) faculties developed between 5 and 7 patient safety topics.

**TABLE 3:** Number of patient safety topics include in the course syllabi. (Spain, 2024).

Number of patient safety topic included	Number of faculties	%
0	7	18
1	4	11
2	9	24
3	8	21
4	6	16
5	1	3
6	2	5
7	1	3
Total	38	100

Regarding the 38 faculties where the course guide was accessed, it was observed that none of them implemented all the recommendations from the WHO’s patient safety curriculum guide, despite 14 years having passed since its publication (Table 3). No faculty covered more than 7 topics recommended by the WHO, while only 1 (3%) faculty covered 7 of the recommended topics in the course guide, and 4 (11%) faculties covered between 5 and 7 patient safety topics. These results underline the limited dissemination of the topics studied, as evidenced by the median of 2 (IQR = 1; 3).

In 21 (56%) medical faculties, between 1 and 3 patient safety topics were developed. The most frequent were Introduction to patient safety, Control of HCAIs, and Patient safety associated with care quality.

In 7 (18%) faculties, none of the WHO’s patient safety curriculum guide topics were detected.

On the other hand, in 22 (58%) of the faculties, patient safety was mainly addressed in the curse syllabus of the Preventive Medicine subject (Table 4), while in 6 (16%) faculties patient safety was addressed in the curse syllabus of the Family Medicine subject.

**TABLE 4:** Courses that include topics on patient safety. (Spain, 2024).

Subject	N	%
Do not have subjects that mention any patient safety topics	**7**	**18**
Healthcare Management	**2**	**5**
Family Medicine	**6**	**16**
Preventive Medicine	**22**	**58**
Public Health	**1**	**3**
**Total**	**38**	**100**

In bold: Number and percentage of each type of courses that include topics of patient safety.

Finally, in 2 (5%) and 1 (3%) medical faculties, patient safety was primarily addressed in the curse syllabus of the Health Management or Public Health subject, respectively.

**FIGURE 1:**
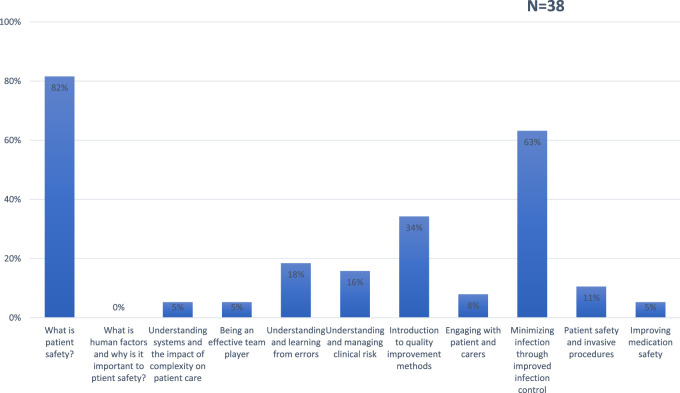
Percentage of topics recommended in the World Health Organization Patient Safety curriculum guide include in medical faculties. (Spain, 2024).

When analyzing the presence of each of the specific topics recommended in the WHO’s patient safety curriculum guide (Figure 1), no medical faculty was identified that specifically addressed the meaning of the “human factor” (the interrelation between the human, their tools, and their work environment) and its importance in patient safety.

Regarding topics related to “understanding systems and the impact of complexity on patient care,” “being a team player” related to patient safety, and “how to improve medication safety,” these are included only by 2 (5%) medical faculties in the curse syllabus of a specific subject.

In 3 faculties (8%), training on “how to engage with patients and care providers” in the context of patient safety training was observed.

In 4 faculties (11%), the topic of “patient safety in invasive processes” was detected.

In 6 faculties (16%), the topic of “how to understand and manage clinical risk” associated with patient safety was identified.

In 7 faculties (18%), the topic on “how to understand and learn from errors in patient safety” was detected.

In 13 faculties (34%), the topic of “quality improvement methods” focused on patient safety was identified.

In 24 faculties (63%), the topic on how to minimize infection through better infection control was detected.

In 31 faculties (82%), the topic about the definition of patient safety was observed.

Finally, the presence of topics related to patient safety was compared based on the type of funding of the university. Specifically, in 5 of the 8 (62.5%) private medical faculties, some topic of patient safety was detected in some subject. In contrast, in 26 of the 30 (86.7%) public medical faculties, some topic related to patient safety was observed in the curse syllabi. These differences are statistically significant (*p* = 0.00) (Table 5).

**TABLE 5:** Presence of Patient Safety as a topic in any course, based on university funding. (Spain, 2024).

		Funding			Fisher’s exact test
		Private	Public	Total	
Patient safety integrated at any subject	No	3 (37.5%)	4 (13.3%)	7 (18.4%)	0.000
	SI	5 (62.5%)	26 (86.7%)	31 (81.6%)	
	Total	8 (100%)	30 (100%)	38 (100%)	

## Discussion

The fundamental principle in medicine, stemming from the Hippocratic Oath and adopted by the World Alliance for Patient Safety since 2004, is “primum non nocere.” This premise underscores the necessity to include patient safety in healthcare education at all educational levels. The results of this study reveal a significant disparity between this expectation and the reality in medical education in Spain. Although 82% of faculties have integrated some patient safety topics, the maximum number of WHO-recommended topics integrated into the training was 7, found in only 1 faculty, and only 11% have integrated between 5 and 7 of the 11 topics recommended by WHO. These results, although they can be inferred based on other studies at both the national and international levels [14, 22, 23, 25–28], are the first obtained with this methodology, in which the training offered in patient safety specifically by medical faculties in Spain is verified.

In our study, we observed progress in the integration of patient safety, as in most medical faculties (82%) there is an introductory topic on patient safety in some subjects. This figure is well above the 45% published in a 2012 study in the United States [23]. Our results are lower than those observed in Japan [14], in a 2012 study, where the implementation rate of patient safety training was 98%. The authors justified this rate by the impetus received from the Japanese Ministry of Education at the time. Although our results may seem acceptable, we must consider the difference in methodology used in evaluating patient safety training, as our study is based on the search in the curse syllabi, while in the mentioned studies, such evaluation was conducted through surveys. Moreover, a very relevant differential factor is the year of evaluation, as there are 10 years of difference between the evaluation in those studies and ours. Therefore, it is possible that the integration of PS in universities in the EU and Japan is currently higher. If we compare ourselves with Europe, more recently in 2023, a study found an integration of patient safety training of 36% in European medical faculties, and 50% in faculties in southern Europe [22], lower values than those found in our study.

In the present study, we did not identify any medical faculty that addresses the 11 topics recommended by the World Health Organization (WHO) in their entirety. This finding is consistent with the study by Jain et al. [23]. However, unlike that study, in which the most frequently addressed topics were infection control, patient transfer, and patient treatment safety, our analysis reveals that, in most cases, the most implemented topics, in addition to the introduction to patient safety, are the control of HCAIs and the introduction to quality improvement methods. This is probably related to the existence of a Preventive Medicine subject in most Spanish medical faculties, which in our study more frequently includes topics related to patient safety. In fact, it is precisely the specialty of Preventive Medicine and Public Health that, with a transversal approach to medicine, develops these topics [29]. As for care quality, in the present study, we observed that 34% of medical faculties integrate this topic into their curse syllabus. This figure is similar to that published in a European study showing that 38% of European medical faculties integrate quality into their curricular plan, with this figure being higher in universities in southern Europe where 50% of the centers integrate quality care topics [22].

Unlike our study, in which some topics were very little addressed or not addressed at all in the curse syllabi of the subjects, such as the human factor or the systemic approach related to patient safety, the study conducted in Japan does describe the importance and integration of these factors, presenting an integration of over 70% in the curse syllabi [14]. On the other hand, the European study did not evaluate the presence of these topics.

In the present study, we detected that patient safety training is offered optionally in 2 faculties. Although probably an attempt to introduce it into the curriculum, it is clearly not sufficient, as it diminishes the relevance of such training, which is considered to be one of the cores of health education [1, 7, 11]. Additionally, it may discourage training in patient safety, a training to which the students themselves assign importance [30].

In our analysis, we found that public university institutions exhibit a significantly higher proportion of patient safety training compared to their private counterparts, a difference that is statistically significant. We found no justification to explain these differences except perhaps the tendency of private faculties to provide training focused on new technologies and topics that excite students more, leaving aside fundamental topics, which *a priori* might seem less attractive.

This finding, along with the known correlation between the phenomenon of “burnout” and the perception of safety culture [31], is relevant for all medical faculties, but even more so for private institutions. This is due to the impact on health professionals’ satisfaction with the interest in health training.

Our study presents several limitations that merit consideration. First, the availability of information regarding the topics covered in the courses varied among faculties, as not all had the corresponding syllabi accessible on their websites. Additionally, there was no established process to verify the information provided on the faculties’ websites. Nonetheless, the absence of patient safety-related topics in these guides implies a potential lack of emphasis in this crucial area. Despite these challenges, exhaustive searches on the faculties’ websites enabled us to collect information from a substantial percentage of them, offering a broad overview of healthcare education in Spain. Furthermore, we addressed the challenge of determining the actual focus of the listed topics by conducting a comprehensive evaluation of the course contents.

The present study has several strengths. It is the first study in our country to evaluate the incorporation of Patient Safety into the educational plans of universities in Spain. The methodology used allows us to gain insight into the state of such education, independent of biases that may be provided by respondents, as seen in other studies and those based solely on students’ knowledge. Furthermore, our findings underscore the urgency of reconsidering the curricular structure in medical faculties to align with the WHO’s recommendations on patient safety training. Achieving this goal necessitates a thorough understanding of the current state of patient safety integration across different medical faculties, underscoring the significance of this study.

In conclusion, according to our findings, the expectations regarding the integration of patient safety education in our setting are not adequately met. Although there is evident progress in incorporating fundamental concepts of patient safety, the comprehensive implementation of all topics recommended by the World Health Organization in medical faculties in Spain is insufficient. These results should serve as a starting point to stimulate a line of research that explores deeply the best strategy to effectively integrate patient safety-related topics into medical education programs.

## Abbreviations

WHO, World Health Organization; PS, Patient Safety; NHS, National Health Service; ANECA, National Agency for Quality Assessment and Accreditation; IRAS, Healthcare-Associated Infections.
