# Supplementary material for: Evaluating the Integration of Patient Safety in Medical Training in Spain
Source: Int J Public Health. 2024 Apr 25;69:1607093. doi: 10.3389/ijph.2024.1607093 (PMC11089277; doi:10.3389/ijph.2024.1607093)
Supplement: Supplementary file 1 [file Table1.docx]

Supplementary File 1: Basic information about medical faculties in Spain. (Spain. 2024)

| **Faculty** | **University Funding** | **University** | **Adress Web** | **Access date** | **City** | **Autonomous Community** |
| --- | --- | --- | --- | --- | --- | --- |
| Faculty of Medicine (Boadilla del Monte) | Private | University CEU San Pablo Madrid | <https://www.uspceu.com/oferta/grado/medicina/plan-estudios> | 14/12/2023 | Madrid | C. Madrid |
| Faculty of Medicine (Madrid) | Public | Autonomous University of Madrid | https://www.uam.es/ss/Satellite/Medicina/es/1234890396092/sinContenido/Estudios_de_Grado.htm | 14/12/2023 | Madrid | C. Madrid |
| Faculty of Medicine and Health Sciences (Alcalá de Henares) | Public | University of Alcalá de Henares | https://www.uah.es/es/estudios/estudios-oficiales/grados/Grado-en-Medicina/ | 14/12/2023 | Madrid | C. Madrid |
| Faculty of Health Sciences (Alcorcón) | Public | King Juan Carlos University | https://gestion3.urjc.es/guiasdocentes/mostrarGuias.jsp# | 14/12/2023 | Madrid | C. Madrid |
| Faculty of Health Sciences (Pozuelo de Alarcón) | Private | Francisco de Vitoria University | https://www.ufv.es/plan-de-estudios-grado-en-medicina/#primer-curso-3 | 14/12/2023 | Madrid | C. Madrid |
| Faculty of Health Sciences (Villanueva de la Cañada) | Private | Alfonso X El Sabio University | [Grado en Medicina. Estudiar Medicina en Madrid - UAX](https://www.uax.com/titulaciones/grado-en-medicina) | 14/12/2023 | Madrid | C. Madrid |
| Faculty of Biomedical Sciences (Madrid) | Private | European University of Madrid | [Carrera de Medicina en Madrid \| Universidad Europea](https://universidadeuropea.com/grado-medicina-madrid/) | 14/12/2023 | Madrid | C. Madrid |
| Faculty of Medicine | Public | Complutense University of Madrid | [Guías docentes, Grado en Medicina, curso 2023-24 \| Facultad de Medicina (ucm.es)](https://medicina.ucm.es/guias-docentes) | 14/12/2023 | Madrid | C. Madrid |
| Faculty of Health | Private | Camilo Jose Cela University | [Estudiar Grado Medicina en Madrid \| UCJC](https://www.ucjc.edu/grados/medicina/) | 14/12/2023 | Madrid | C. Madrid |
| Faculty of Medicine (Sant Joan d'Alacant) | Public | Miguel Hernández University of Elx | [UMH - Grado en Medicina - Plan de Estudios](https://www.umh.es/contenido/Estudios/:tit_g_132_M1/datos_es.html?frm=pintaPlanEstudios) | 14/12/2023 | Elche | C. Valenciana |
| Faculty of Medicine and Dentistry (Valencia) | Private | Catholic University of Valencia San Vicente Mártir | [Medicina \| UCV](https://www.ucv.es/oferta-academica/grados/grado-en-medicina/seccion/plan-de-estudios) | 14/12/2023 | Valencia | C. Valenciana |
| Faculty of Medicine and Dentistry (Valencia) | Public | University of Valencia | <https://www.uv.es/uvweb/grado-medicina/es/se-estudia/plan-estudios/plan-estudios/grado-medicina-1285938467926/Titulacio.html?id=1285847387054&plantilla=GRAU_Medicina/Page/TPGDetaill&p2=2> | 14/12/2023 | Valencia | C. Valenciana |
| Faculty of Health Sciences (Moncada) | Private | CEU Cardenal Herrera University | <https://www.uchceu.es/estudios/grado/medicine/plan-estudios> | 04/01/2024 | Valencia | C. Valenciana |
| Faculty of Health Sciences (Castellón) | Public | Jaume I University | <https://ujiapps.uji.es/sia/rest/publicacion/2023/estudio/235> | 04/01/2024 | Valencia | C. Valenciana |
| Faculty of Health Sciences | Public | University of Alicante | <https://web.ua.es/es/grados/grado-en-medicina/plan-de-estudios.html> | 04/01/2024 | Alicante | C. Valenciana |
| Faculty of Medicine (Cádiz) | Public | University of Cádiz | [https://medicina.uca.es/docencia/grado-en-medicina/planes-de-estudios-itinerario-curricular/ https://asignaturas.uca.es/asig/](https://medicina.uca.es/docencia/grado-en-medicina/planes-de-estudios-itinerario-curricular/) | 04/01/2024 | Cadiz | Andalucia |
| Faculty of Medicine (Granada) | Public | University of Granada | <https://www.ugr.es/estudiantes/grados/grado-medicina> | 04/01/2024 | Granada | Andalucia |
| Faculty of Medicine (Málaga) | Public | University of Málaga | <https://www.uma.es/grado-en-medicina/cms/menu/informacion-grado/competencias/> | 04/01/2024 | Malaga | Andalucia |
| Faculty of Medicine (Sevilla) | Public | University of Seville | <https://www.us.es/estudiar/que-estudiar/oferta-de-grados/grado-en-medicina#edit-group-plani> | 04/01/2024 | Sevilla | Andalucia |
| Faculty of Medicine and Nursing (Córdoba) | Public | University of Córdoba | https://www.uco.es/organiza/centros/medicinayenfermeria/es/grados/gr-medicina#resultados | 04/01/2024 | Córdoba | Andalucia |
| Faculty of Health Sciences | Public | University of Almeria | <https://www.ual.es/estudios/grados/presentacion/plandeestudios/3321> | 04/01/2024 | Almeria | Andalucia |
| Faculty of Medicine | Public | University of Jaen | <https://www.ujaen.es/estudios/oferta-academica/grados/grado-en-medicina#informacion-academica> | 04/01/2024 | Jaen | Andalucia |
| Faculty of Medicine (Cerdanyola del Vallès) | Public | Autonomous University of Barcelona | <https://www.uab.cat/web/estudiar/listado-de-grados/plan-de-estudios/guias-docentes-1345467893062.html?param1=1263281708763> | 24/01/2024 | Barcelona | Cataluña |
| Faculty of Medicine and Health Sciences (Lleida) | Public | University of Lleida | <https://graumedicina.udl.cat/es/pla-formatiu/pla-estudis-guies-docents/> | 24/01/2024 | Lleida | Cataluña |
| Faculty of Medicine (Barcelona) | Public | University of Barcelona | <https://www.ub.edu/portal/web/medicina-ciencies-salut/graus/-/ensenyaments/detall/4798347/27> | 24/01/2024 | Barcelona | Cataluña |
| Faculty of Medicine and Health Sciences (Sant Cugat del Vallès) | Private | International University of Catalonia | <https://www.uic.es/es/estudis-uic/medicina-y-ciencias-de-la-salud/grado-en-medicina#plan-de-estudios-y-equipo-docente> | 24/01/2024 | Barcelona | Cataluña |
| Faculty of Health and Life Sciences (Barcelona) | Public | Pompeu Fabra University | <https://gestioacademica.upf.edu/doa/consultaPublica/look%5Bconpub%5DBuscarPubGuiaDocAs?entradaPublica=true&idiomaPais=ca.ES&_centro=336&_estudio=3363&_anoAcademico=2021> | 24/01/2024 | Barcelona | Cataluña |
| Faculty of Medicine and Health Sciences (Reus) | Public | Rovira i Virgili University | <https://www.fmcs.urv.cat/es/estudios/medicina/> | 24/01/2024 | Reus | Cataluña |
| Faculty of Medicine (Girona) | Public | University of Girona | <https://www.udg.edu/es/estudia/oferta-formativa/oferta-dassignatures?idpla=3109G0111&anyacad=2023> | 24/01/2024 | Girona | Cataluña |
| Faculty of Medicine | Private | Vic-Central University of Catalonia | <https://www.uvic.cat/grau/medicina> | 24/01/2024 | Barcelona | Cataluña |
| Faculty of Health Sciences (Guadalupe) | Private | Catholic University San Antonio of Murcia | <https://www.ucam.edu/estudios/grados/medicina-presencial/plan-de-estudios> | 24/01/2024 | Murcia | Murcia |
| Faculty of Medicine | Public | University of Murcia | <https://www.um.es/web/estudios/grados/medicina/plan-guias> | 24/01/2024 | Murcia | Murcia |
| Faculty of Medicine and Dentistry (Santiago de Compostela) | Public | University of Santiago de Compostela | <https://www.usc.gal/es/estudios/grados/ciencias-salud/grado-medicina> | 24/01/2024 | Santiago de Compostela | Galicia |
| Faculty of Medicine (Santander) | Public | University of Cantabria | <https://web.unican.es/estudios/detalle-estudio?pi=66&a=2023> | 24/01/2024 | Santander | Cantabria |
| Faculty of Medicine (Badajoz) | Public | University of Extremadura | <https://www.unex.es/conoce-la-uex/centros/medicina/titulaciones/info/competencias?id=0212> | 01/02/2024 | Caceres | Extremadura |
| Faculty of Medicine (Pamplona) | Private | University of Navarra | <https://www.unav.edu/web/grado-en-medicina/plan-de-estudios#:~:text=Gu%C3%ADa%20Acad%C3%A9mica%2020/21%3A%20Tablet%20y%20PC> | 01/02/2024 | Navarra | Navarra |
| Faculty of Medicine | Public | Public University of Navarra | <https://www.unavarra.es/sites/grados/salud/medicina/presentacion.html> | 01/02/2024 | Navarra | Navarra |
| Faculty of Medicine (Tenerife) | Public | University of La Laguna | <https://www.ull.es/grados/medicina/> | 01/02/2024 | Tenerife | Islas Canarias |
| Faculty of Health Sciences (Las Palmas de Gran Canaria) | Public | University of Las Palmas de Gran Canaria | <https://www2.ulpgc.es/plan-estudio/4029> | 01/02/2024 | Gran Canaria | Islas Canarias |
| Fernando Pessoa University Canarias | Private | University in Gran Canaria | <https://www.ufpcanarias.es/G1G022> | 01/02/2024 | Gran Canaria | Islas Canarias |
| Faculty of Medicine and Dentistry (Leioa, Vizcaya) | Public | University of the Basque Country | <https://www.ehu.eus/es/web/graduak/grado-medicina/creditos-y-asignaturas> | 01/02/2024 | Pais Vasco | País Vasco |
| Faculty of Health Sciences (Deusto) | Private | Deusto University | <https://www.deusto.es/es/inicio/estudia/estudios/grado/medicina> | 01/02/2024 | Pais Vasco | País Vasco |
| Faculty of Medicine (Valladolid) | Public | University of Valladolid | <https://acesse.dev/1nwLs> | 01/02/2024 | Valladolid | Castilla y Leon |
| Faculty of Medicine (Salamanca) | Public | University of Salamanca | <https://facultadmedicinaorg.files.wordpress.com/2020/07/plan-de-estudios_medicina-boe-24-10-2016.pdf> | 01/02/2024 | Salamanca | Castilla y Leon |
| Faculty of Medicine (Ciudad Real) | Public | University of Castilla-La Mancha | <https://www.uclm.es/estudios/grados/medicina> | 01/02/2024 | Ciudad real | Castilla La Mancha |
| Faculty of Medicine (Albacete) | Public | University of Castilla-La Mancha | <https://www.uclm.es/albacete/medicina/gradomedicina/informaciongrado> | 01/02/2024 | Albaceta | Castilla La Mancha |
| Faculty of Medicine (Zaragoza) | Public | University of Zaragoza | <https://estudios.unizar.es/estudio/asignaturas?anyo_academico=2023&estudio_id=20230118&centro_id=104&plan_id_nk=304&sort=curso> | 01/02/2024 | Zaragoza | Aragon |
| Faculty of Medicine (Oviedo) | Public | University of Oviedo | <https://www.uniovi.es/estudia/grados/salud/medicina> | 01/02/2024 | Oviedo | Asturias |
| University of the Balearic Islands | Public | University of the Balearic Islands | <https://estudis.uib.es/digitalAssets/382/382197_DEF-May-PBlava-GMED.pdf> | 01/02/2024 | Islas baleares | Islas Baleares |
